# Options for states to constrain pricing power of health care providers

**DOI:** 10.3389/frhs.2022.1020920

**Published:** 2022-10-19

**Authors:** Katherine L. Gudiksen, Robert B. Murray

**Affiliations:** ^1^The Source on Healthcare Price and Competition, University of Hastings College of the Law, San Francisco, CA, United States; ^2^Global Health Payment LLC, Towson, MD, United States

**Keywords:** consolidation, provider prices, rate review, rate restriction, affordability, rate setting, cost constraint, market failure

## Abstract

Health care is becoming increasingly unaffordable for both individuals and employers and prices vary in nearly incomprehensible ways that do not correlate with quality. In many areas, consolidation of insurers and providers resulted in market failure that needs policy interventions. With federal gridlock, state policymakers are seeking options for controlling health care costs in markets where competition has failed. In this article, we discuss a spectrum of options that policymakers have to more directly control healthcare prices: (1) establishing a cost-growth benchmark, (2) creating a public option, (3) capping or establishing a default out-of-network payment rate for health care services, (4) creating affordability standards that authorize the insurance commissioner to reject contracts with excessive rate increases, (5) creating global budgets for hospital-based care, (6) capping excessive prices and/or tiering allowed rate updates, and (7) creating a population-based payment model. We provide a roadmap for state policymakers to consider these options, review the experiences with states who have tried these models, and discuss additional design considerations that policymakers should consider with any of these models. In the 1970's and 1980's, during a time of rapid growth in health care prices and spending, states took a decisive leadership role in developing regulatory models to curb the growth in health care costs and improve affordability for their citizens. It is time for states to lead the nation once again in addressing the current health care cost and affordability crisis in the U.S.

## Introduction

Health care spending now accounts for nearly one-fifth of the U.S. economy (1), and there are numerous indications that many healthcare markets are not operating efficiently—prices for health care have grown much faster than inflation (2), prices for commercially-insured patients have grown much faster than those for Medicare and Medicaid (3), and these higher prices do not correlate with higher quality (4, 5). A primary driver of these market problems is the rapid and unrelenting consolidation of providers into large health systems (6). Additionally, the Government Accountability Office found that 43 states had highly concentrated insurance markets for individual, small group, and large group plans (7). In markets where insurers have the bargaining power to reduce prices, research shows that any savings from lower health care prices are not passed on to consumers and employers (8, 9). When both the provider and insurer markets are highly concentrated, the dominant providers and insurers can extract and divide excess profits that result from their combined market power. While increased antitrust enforcement (at both the state and federal level) may slow future consolidation, this enforcement does little to address the market power of extant health care monopolies (4, 10).

Policymakers at all levels are looking for a plan. Congress considered legislation to restrict anticompetitive conduct and promote competition in healthcare markets (11), but political polarization at the federal level means that comprehensive action to address healthcare costs is unlikely to pass. Consequently, state policymakers should step into the void left by federal inaction and consider policy options to restrain excessively high and rising healthcare prices and to improve overall affordability of health care. States have authority to regulate health insurance and the provision of health care within their borders and are large purchasers of healthcare services through state Medicaid, CHIP, and state employee health benefit plans. State policymakers may also be more successful than their federal counterparts at bringing together diverse stakeholders to work on policy solutions (12). Additionally, state lawmakers may be more agile and better able to adjust policy solutions over time than those passed by Congress. Finally, in many cases, the most effective policies respond to distinct market characteristics and market actors, so tailored approaches may be more successful than a universal federal approach (12). Therefore, state policymakers may be uniquely positioned to assess the underlying reasons for current market dysfunction and design policies to target specific market failures.

In recent years, a handful of states have made initial efforts to regulate prices that offer promise, but most have fallen short of their potential. This paper reviews those efforts and offers guiding considerations and best practices for state policymakers. We comprehensively searched legislation in all 50 states and spoke to experts in many states to develop a list of state efforts to control provider prices in the last decade. We group these efforts into a spectrum of options and provide a roadmap for state policymakers to begin addressing provider prices. We discuss seven policy options ranging from those requiring the least amount of regulatory intervention and having the lowest direct impact on prices and expenditures to those with the tightest control on overall expenditures: (1) establishing a cost-growth benchmark, (2) creating a public option, (3) capping or establishing a default out-of-network payment rate for health care services, (4) creating affordability standards that authorize the insurance commissioner to reject contracts with excessive rate increases, (5) creating global budgets for hospital-based care, (6) setting prices, capping excessive prices, and/or tiering allowed rate updates, and (7) creating a population-based payment model. These options provide state policymakers with ways to control health care price and expenditure growth that has been effective and/or can feasibly be implemented at a state level, depending on the specific political and health market dynamics in a given state. Several of our proposed models, including global budgets and population-based payment models, also have the ability to control the growth of health care expenditures caused by increased health care volumes.

The broad range of options means that policy solutions can be tailored to specific market conditions, political climate, and ideology of the state, but an array of options risks decision paralysis, where legislators can endlessly discuss policy options and design choices. To combat that potential risk, this article provides a roadmap to facilitate strategy formulation and considerations for state policymakers when choosing among the options. Depending on market conditions, a combination of mutually enforing models may be most effective. Consistent with the concept of a continuum of options, state policymakers should begin with whichever model is most immediately feasible and pivot or augment their initial approach in future years.

## Provider rate regulation implementation roadmap

The first step on the path to provider rate regulation is to evaluate whether policymakers have sufficient, high-quality data about health care costs and expenditures to answer core questions about the performance of a state's health care system and its cost drivers. In the thirty states with an all-payer claims database (APCD), policymakers likely have access to inpatient, outpatient, physician, and/or prescription drug claims data (13, 14). Some states also collect patient cost-sharing and quality information that is key to evaluating overall costs. States may use additional, federal data sources, like the national spending account data, published by the Centers for Medicare and Medicaid Services (CMS) (15), data from the Health Care Cost Institute (16), and the RAND Hospital Price Transparency Study (17) to both validate data and fill in gaps in state data, especially in states without mandatory APCDs.

Unfortunately, the data from national sources and state-based APCDs may not be robust enough for policymakers to answer detailed questions. Some states only collect data from private plans and exclude public payers (13). Also, following the Supreme Court ruling in *Gobeille v. Liberty Mutual* [136 S. Ct. 936 (18)], states can no longer compel self-funded plans – those in which the employer retains the financial risk for paying for health care services – regulated by the Employee Retirement Income Security Act (ERISA) (19) to submit claims data to APCDs. While many employers voluntarily submit data to state APCDs, the lack of mandatory submission means that in some states, the claims for less than a quarter of the lives covered by self-insured plans may be reported to the APCD (13). Even in states where a large number of ERISA plans choose to participate, data may be skewed with respect to plan offerings, enrollment, or health care prices as employers offering more comprehensive coverage may be overrepresented or employers that previously reported data to an APCD may stop reporting if they reduce health care coverage (13). Furthermore, APCDs may not capture non-claims spending like provider performance incentive payments and prospective payments for health care services (e.g., capitation) (20). Additionally, few states collect data for alternative payment models (APMs), like patient-centered medical homes and accountable care organizations (ACOs), particularly commercial ACOs, and even states that do collect that data may find it difficult to compare fee-for-service (FFS) claims data with that from the APMs (21). Many researchers and government agencies are working to improve the comprehensiveness of APCD data, and an advisory committee at the Department of Labor made recommendations, including key factors in a standard data layout, to encourage participation by self-funded employers in state APCDs (22). Nevertheless, states wanting to adopt a cost-growth benchmark likely need to supplement data collected from payers in an APCD with additional detailed and disaggregated data to identify factors driving cost growth (23). Policymakers can supplement APCDs with hospital discharge data, payer expenditure reports, provider financial reports (24), and publicly available audited financial statements for non-profit healthcare systems (25).

In addition to cost data, states desire data about quality, but there are general concerns about how robust quality measures are. For example, publicly available hospital quality rating systems frequently offer conflicting results (26), and a recent study found that Medicare Hospital Compare star ratings were highly sensitive to how performance ratings are calculated, with no compelling methodology for measuring hospital performance (27). While policymakers should strive to measure quality, pressing concerns about health care affordability suggest that lawmakers should move forward in addressing costs, even in the absence of robust quality data.

Once policymakers have sufficient, representative data, they can evaluate prices, utilization, and cost-growth on a regional and payer- and provider-specific basis to identify cost drivers and particular geographies and specialties where markets appear to have failed. Based on this analysis, policymakers can develop a list of policy goals and priorities, including those that target problematic provider sectors or payers.

### Step 1: Consider a cost-growth benchmark

Many policymakers will likely want to begin by considering a cost-growth benchmark (CGB). A CGB is a per annum target for the state's rate of growth for total spending on health care, including payments made by public and private payers and patients (28). CGBs authorize state officials to measure the total cost of health care in the state over time and anchor public expectations for increases in health care costs. Policymakers can then compare total spending on health care each year to the cost growth target and conduct data analyses to uncover specific cost growth drivers, including specific payers, health systems, or provider groups (29). When establishing a benchmark, policymakers aim to keep total spending on health care from growing faster than the economy or wages, so most states set a benchmark that considers measures of economic growth (i.e., the potential gross state product, PGSP).

Unlike the other policy interventions to address provider rates, CGBs can be established without legislative action (30). A governor may establish a CGB by directing an existing state agency, like the one overseeing a state APCD, to consider healthcare spending and set a target for the state without requiring any legislative action (31). CGBs created by executive order are essentially a voluntary target as they typically do not include financial penalties for non-compliance. While voluntary targets may seem less effective than those with financial penalties, methodological difficulties in accurately measuring and enforcing the cost growth performance of individual provider entities make it unclear whether financial penalties can be assessed in a way that meaningfully reduces healthcare spending (32, 33). As a result, policymakers may find that the implementation of a CGB to be the most politically feasible initial approach.

#### State experiences with CGBs

Massachusetts created the first CGB in 2012, and seven more states adopted CGB programs between 2018 and 2021 (24). It is too early to evaluate the effectiveness of the CGBs in most states, but experts credit the CGB and other cost savings measures in Massachusetts with keeping health care spending growth at or below the national average for 10 consecutive years, saving an estimated $7.2 billion cumulatively between 2013 and 2019 (34). While this early attempt at moderating health care spending growth may have persuaded providers to temporarily moderate price increases, this early success appears to be waning. In a report analyzing data from 2012 to 2019, the Health Policy Commission (HPC) notes that “[d]espite several years of notable progress, spending has grown in excess of the benchmark for the past 2 years” (35). Massachusetts law allows the HPC to require payers and providers with excessive spending growth to implement a performance improvement plan (PIP) and impose a fine of up to $500,000 (36), but the HPC did not require any PIPs in the first decade after establishing the CGB. In January 2022, the HPC voted to require Mass General Brigham (MGB) to implement the first PIP, but the maximum penalty is <0.2% of the estimated excessive charges of $293 million in cumulative commercial cost-growth above the benchmark (37), leading experts to remain skeptical that the CGB can be an effective tool to limit provider rates in Massachusetts.

Oregon attempted to improve on the Massachusetts law by requiring PIPs with an escalating accountability mechanism that includes “meaningful financial penalties” for any provider or payer that exceeds the cost-growth target with statistical confidence without reasonable cause in three out of five calendar years or for two consecutive years (38). In 2022, California created the Office of Health Care Affordability that also attempts to impose meaningful penalties for excessive spending over a benchmark (39). These larger penalties might make the CGB in these states more effective.

Fundamental limitations in both data collection and analysis, however, may render CGBs relatively ineffective as a single tool to reduce healthcare costs. For instance, regulators likely want to risk-adjust the benchmark when applying it to specific payers or providers to account for changes in illness severity over time (40). Yet, risk-adjustment methodologies can be susceptible to more aggressive provider documentation and coding practices that may inflate risk scores relative to a base year. Specifically, increased documentation and coding of co-morbidities/secondary diagnoses for each patient may lead to higher risk scores that may not reflect an actual deterioration of the health status of that population (41). Rising payer risk scores in Rhode Island in 2018 and 2019 effectively doubled the cost-growth target for payers with an average rising risk score and Massachusetts observed steadily rising risk scores, amounting to an 11.7% increase, between 2013 and 2018 (42). Consequently, the HPC recommended evaluating performance on a non-risk adjusted basis (35). Furthermore, to measure whether specific providers or provider groups meet a total cost of care benchmark, regulators likely need to track spending for a particular patient and then attribute the cost or portion of the cost of their care to a provider organization (43). While insurers routinely make these attributions for value-based care contracts, such a process can be much more difficult to do statewide, especially for enrollees in PPO plans.

All of these shortcomings mean that the use of CGB may provide a short-term sentinel constraint on price and expenditure growth, but the methodological difficulties associated with enforcing compliance with the growth targets at an individual provider level may render this approach ineffective over the longer term. Nonetheless, CGBs can lead to increased price transparency and improve understanding of the drivers of health care spending in the state. For example, the 2021 HPC report found that an increase in prices was the primary reason that spending exceeded the benchmark in Massachusetts in 2018 and 2019, but that an increase in utilization also contributed, especially because 70% of the growth for outpatient hospital visits occurred at academic medical centers rather than less expensive community hospitals (35). Understanding these drivers led the HPC to recommend that the state make health plans more accountable for excessive spending, consider implementing more direct rate-setting models to constrain excessive provider prices and price growth, and improve payment equity, particularly for providers serving high proportions of publicly insured patients. To fulfill these targets, the HPC specifically recommended the Massachusetts legislature consider passing other models discussed in this paper, including price caps on out-of-network care, affordability standards, and price caps on the highest-priced providers.

#### Considerations for states implementing CGBs

While CGBs may be fundamentally limited by the difficulty in quantitative risk adjustment and the ability to attribute the cost of patient care to individual providers, establishing a CGB may give policymakers compelling evidence to identify specific policy targets, push states to develop a data/intelligence infrastructure sufficient to give the state a more informed strategy moving forward, and help coalesce diverse stakeholders in support of additional cost-controlling measures. CGBs may also be less effective at controlling costs than other options as they are retroactive—at best, they make providers accountable for past spending. Nonetheless, CGBs may be more easily implemented than other models discussed in this report and can be designed to expend few state resources.

### Step 2: Evaluate policy options and the potential for regulatory oversight

Once policymakers have sufficient data to identify the drivers of state health spending, they should consider whether they want policies to control *price growth* or overall *expenditure growth*. High and rising prices are a major driver of the increasing unaffordability of health care, particularly in the hospital sector. Thus, policymakers may choose to focus specifically on regulatory models that constrain prices. However, hospitals paid on an itemized FFS basis (per case, per visit, per ancillary test) have cost structures that allow them to increase profit margins by increasing the volume of services they provide. For example, in the early 2000's, the Maryland rate regulatory system exerted tight controls over hospital price updates but found that hospitals dramatically increased the volumes of services they provided, allowing hospitals to *increase* their profitability and increase overall hospital spending despite limits on price increases (44). These incentives are consistent with the FFS incentives of physicians to expand service volumes and can be amplified by stringent limits on provider price growth. Consequently, policymakers should consider whether they have the resources and expertise to monitor increases in volume of service, in particular the provision of low value and marginal services, and whether they have the infrastructure (or political will and resources to create the infrastructure) to implement budget-based payment models or volume adjustment systems.

Second, policymakers should ask if large price disparities exist among providers in the state, and if narrowing those disparities is a priority for policymakers. Large variations in prices can indicate market failure and distorted prices send misguided signals that result in under- and over-utilization of some healthcare services (10). Price disparities can also result in lower overall payments for less desirable (“have not”) hospitals, which can compromise quality, especially when the payments are below marginal cost (10). In Massachusetts, the Attorney General's Office (AG) found large disparities in the prices paid to hospitals and provider groups and concluded that the differences in payment were primarily due market power, brand name, and facility location (45). The AG's report further found that dominant provider systems use contracting practices that reinforce and perpetuate these disparities in ways that can undermine both access to and the quality of care at underpaid facilities, resulting in a two-tiered system of health care delivery. Consequently, policymakers may want to prioritize models that reduce disparities by applying smaller annual price updates for high-priced providers, especially those with market power, while allowing low-priced providers to increase rates more rapidly. Policymakers could also consider establishing floors on negotiated prices to protect hospitals from dominant insurers that aim to push rates below average cost and to protect rural and safety-net providers.

Once policymakers have considered these guiding questions, they should consider developing a regulatory framework that can address the identified issues and adequately evaluate the performance of that model in controlling both prices and expenditures. On the spectrum of options presented here, three models have a light regulatory touch (i.e., less regulatory oversight requiring less methodological complexity)–a public option, caps on OON services, and affordability standards–and three–global budgets, price caps and price growth limitations, and population-based models–require more regulatory oversight.

### Step 3: Evaluate “light touch” regulatory intervention models

Many states are unlikely to have the necessary data, resources, political environment, and stakeholder engagement to immediately pursue the more robust models we present below and may want to adopt a staged approach that starts with one or more of the “light touch” models and then progress to more comprehensive models as they gain experience. Accordingly, states may choose lighter touch models because they can often be implemented without establishing a new regulatory agency. Additionally, legislators could consider developing bills to implement all three of these light touch approaches simultaneously as they are likely to be mutually reinforcing. Doing so would also potentially enable legislators to negotiate political compromises to increase the potential for any one of these options to pass.

#### Option 1: State public option plans

A state public option plan is a state-initiated insurance plan offered to a significant share of the private health insurance market that pays providers publicly determined rates (46). State public options typically set premiums to cover average medical expenditures and can grant residents access to state or federal subsidies when purchasing coverage. If the public option provides a lower cost insurance option than existing private plans, and individuals and employers view the public option as an attractive alternative to private insurance, private plans risk losing market share to the public option. This competition could increase the negotiation leverage that insurers have when establishing networks, which could cause prices for healthcare services to converge toward those paid by the public option (47). However, regulators designing a public option face a difficult balancing act–the public option needs to have prices high enough to develop a broad network of providers but low enough to generate cost savings and attract consumers to the public option. As a result, policymakers face trade-offs when designing a public option plan. They must either mandate provider participation at lower prices and risk political opposition or set provider payment rates high enough to encourage participation, limiting the public options' effectiveness at controlling costs. Furthermore, if states do not mandate provider participation, some experts warn that a public option could lead to higher provider prices in the commercial market if dominant providers are able to demand even higher prices from private insurers either because those remaining in private insurance are less price sensitive or because a loss of profits from the public option drives additional consolidation among providers (48).

##### The experience of states with public option plans

Three states–Washington, Nevada, and Colorado – passed laws requiring private insurers to sell public option plans (S.B. 5526, 66th Legis., 2019 Reg. Sess. (Wash. 2019); H.B. 21-1232, 73rd Gen. Asemb., 1st Reg. Sess. § 6 (Colo. 2021); S.B. 420, 2021 Legis., 81st Reg. Sess. (Nev. 2021). None of the states mandated participation by all providers, but Nevada's law comes the closest by requiring providers that participate in Medicaid or the state employee health benefits plan (SEHBP) also participate in at least one public option plan (S.B. 420 § 13). Washington did not initially mandate any participation, but subsequently revised the law to require the participation of hospitals that accept payment from Medicaid or the SEHBP and are in a county in which no public option is currently for sale (S.B. 5526 (2019), S.B. 5377 (2021). Finally, Colorado allows the Insurance Commissioner to require hospitals to participate in the public option at rates determined by the Department of Insurance (DOI), if it is “necessary to ensure the standardized plan meets the premium rate requirements and the network adequacy requirements” (Colo. Rev. Stat. § 10-16-1306 4(c)).

Washington's law specifies an aggregate cap on payments to hospitals and physicians at 160% of Medicare rates, which was below the current estimated statewide commercial price average of 174% of Medicare rates (49). Alternatively, Nevada and Colorado require private insurers to sell public option plans and reduce premiums by at least 5% in the first year, but they let industry players decide how to achieve these cuts initially. If private negotiations fail to effectively control costs, the states can then assume greater control, through direct administration of the public option in Nevada and hospital rate-setting in Colorado (50). While Nevada's regulatory backstop is somewhat vague, in Colorado, if private insurers cannot reach the premium targets and meet state network adequacy standards, the DOI may hold public hearings and order providers to participate in the public option at DOI-established payment rates. Colorado and Nevada aim to begin selling plans in 2023 and 2026, respectively, so whether their public options can effectively control healthcare costs is unknown. In Washington, the public option was not significantly cheaper than existing insurance options in the first year it was sold (51), so lawmakers considered subsequent legislation to increase the affordability of the program. Initial versions of the bill would have reduced reimbursement rates to 135% of Medicare rates for most hospitals (*S.B. 5377* 2021), but ultimately, lawmakers were unsuccessful at reducing hospital payment rates in the public option (49). This experience emphasizes the struggle lawmakers may face when trying to reduce provider payment rates and place downward pressure on commercial rates.

##### Considerations for states implementing public options

Policymakers assessing whether a public option is an appropriate model for their state should assess competition in both provider and insurer markets. The public option may be most effective in states with limited insurance choices, especially those with few insurers offering plans on the exchange. In those states, the public option may increase competition among plans and provide affordable insurance in all areas of a state (46). Additionally, a public option may increase the effectiveness of other provider rate-setting models. Without competition among insurance plans, insurers may be able to retain some of the savings from lower provider rates as employers and consumers do not have a viable lower-cost insurance option (8, 9). Consequently, a public option can increase competition among plans and give insurers a financial incentive to pass savings on to consumers or risk losing market share.

States looking to create a public option can learn from these forerunner states as all three took different routes when balancing provider participation and prices. If policymakers choose to allow health systems with high reputations, like academic medical centers, to opt out of the public option, the attractiveness of this model will likely decrease and policymakers risk creating a two-tiered system. In areas with a few high-priced providers, policymakers may follow Colorado's lead of authorizing the DOI to hold hearings and mandate participation of these providers at state-determined rates. Learning from Washington's experience, states may choose not to set provider rates in statute as decreasing those rates can be difficult and require repeat legislative action where lawmakers must withstand strong lobbying efforts from the well-funded provider organizations. Placing rate-setting authority in a state agency, like in Colorado, may hold more promise to control costs. An agency is likely more agile than the state legislature at responding to specific market inefficiencies by imposing constraints on the highest provider rates and at identifying counterproductive industry practices like upcoding or the use of incentive payments that may increase overall costs. Concentrating all of the rate-setting authority in an agency, however, risks regulatory capture, in which a regulated industry unduly influences regulators to satisfy its interests rather than the public interest, or regulatory failure, in which complexity and inability to accommodate innovations in health care delivery and payment undermine the agency (52).

#### Option 2: Caps on OON rates

The second low-intensity model would create state-administered caps on out-of-network (OON) prices paid by commercial insurers. When using OON caps, the state sets a maximum payment that insurers pay when a patient obtains care from a provider outside their insurance network. OON caps could be applied to all out-of-network services or limited to “surprise billing” situations^.^ Surprise bills occur when patients unknowingly get care from providers that are out of their insurance plans' network, including emergency situations, or when patients are seen by a provider that is out-of-network at an in-network facility (53). OON price caps can generate savings by truncating very high OON price levels and more importantly, have an indirect spill-over effect on in-network negotiated rates. With OON caps, insurers should be able to negotiate in-network rates that are close to the OON price cap because, if the provider refused to contract near the OON rate, the insurer could cancel the provider's contract and simply pay the capped price for all OON services delivered by that provider (54, 55). The OON price cap is a “lower-intensity” regulatory approach because it applies regulatory intervention to a relatively small segment of services (around 6.1% for professional and lab services) (56) and even less for hospital services (48), leaving providers and insurers to negotiate over in-network rates without additional regulatory intervention. Furthermore, unlike the price and rate update model discussed later, OON caps are compatible with alternative payment models (APMs) or risk-sharing arrangements because OON caps only apply when there is no existing contract between an insurer and provider.

##### Examples of state and federal caps on OON prices

At the federal level, the Social Security Act (57) requires OON providers serving Medicare Advantage (MA) beneficiaries to accept the Medicare FFS rate as full payment for any services provided. If the parties cannot reach an agreement in contract negotiations on acceptable in-network rates, this requirement means that providers will remain out-of-network and be required to bill no more than 100% of FFS Medicare rates. This statutory provision created a de facto OON cap on MA rates for most physician and hospital services. In practice, this provision gives MA plans increased negotiating leverage over most providers and results in in-network negotiated MA plan payments that in most cases closely approximate FFS Medicare rates (55).

There are limited exceptions to this negotiation dynamic in the MA market. For instance, in the market for dialysis services, two entities have approximately 80% market share (58). This dominant market position along with network adequacy requirements imposed by the MA program, appear to have allowed these providers to negotiate MA rates that are more than 114% of FFS Medicare rates (58, 59). While MA negotiated fees to dialysis providers are higher than other MA negotiated in-network rates, the presence of the de facto OON price cap appears to attenuate the ability of dominant providers to demand exorbitant commercial in-network price levels, which are concentrated in the range of 150 to 240% of Medicare FFS rates (3). Medicare removed dialysis clinics from the list of providers subject to network adequacy requirements in MA plans in June 2020 (60), but it remains unknown if in-network MA negotiated prices dropped as a result.

The experience in the MA market provides evidence of how such price caps may work in commercial markets, but the MA experience may not be fully generalizable to the commercial market. One major difference is that hospitals may use much higher commercial rates as a “safety valve” to enable them to accept in-network MA rates close to traditional Medicare (55). Accordingly, some have speculated that “must-have” providers, (i.e., highly desired providers and/or that insurers need to have in their network to offer a commercially viable plan) may be able to demand in-network commercial prices higher than an OON price cap because employers may insist that these providers be included in-network to satisfy the demands of their employer accounts (61). Medicare and state network adequacy requirements can exacerbate this problem by requiring plans to have an adequate number of providers in a network (60, 62). Nonetheless, the de facto OON cap appears to have conferred negotiating leverage to MA plans that has likely generated significant savings relative to their current commercially negotiated in-network price levels (55). While the MA experience with OON caps is instructive, how OON price caps might influence in-network negotiated rates in the commercial sector remains unknown.

At the state level, excluding the direct rate-setting programs of the 1970's and 80's, no state has implemented OON price caps for all services. Some states and the federal government, however, have imposed limits on OON provider bills for surprise bills (63). The federal No Surprises Act protects patients by limiting their cost sharing when they receive emergency care, some post-stabilization services, and non-emergency services at in-network facilities and uses an independent dispute resolution (IDR) process to resolve payment disputes between payers and OON providers. The Congressional Budget Office projected that an IDR process that presumed the payment rate should be the median in-network rate in a geographic area would case OON payments to converge around in-network median rates (64). Some states also set payment standards for surprise bills. For example, California sets a payment standard of the greater of (1) 125% of Medicare FFS rates or (2) the average contracted rate for that health plan and for that region for non-emergency services provided by OON providers at in-network facilities (65). LaForgia et al. found that prices paid to both in-network and OON anesthesiologists for out-patient care decreased after the adoption of California's payment standards, suggesting a strong spillover effect (66).

##### Considerations for states implementing OON price caps

As no state has yet adopted OON caps for all services, policymakers should be aware of at least four potential limitations of OON caps. First, in non-emergency situations, OON providers may refuse care for patients if the OON cap is set too low (47). Federal law requires hospitals to provide emergency and stabilization care (67), but providers may restrict care in other situations. For example, in February 2022, the Mayo Clinic in Minnesota announced that they will no longer schedule appointments for OON MA patients unless federal law requires its physicians to care for them (68). Nonetheless, unless a hospital is capacity constrained and can replace OON patients with higher paying in-network patients or the OON cap is set below the marginal cost of care, hospitals will still make an incremental profit by providing OON care. Second, while OON price caps may help reduce in-network negotiated rates, the use of an OON cap may impose a trade-off in the form of reduced provider networks depending on the level of the OON price cap. For instance, a recent study found that a reduction in OON prices by 50% might reduce the share of hospitals participating in insurer networks by 15 percentage points (69). Third, as noted above, OON caps may be less effective in markets with “must-have” providers. Despite the existence of an OON price cap, employers may demand, or network adequacy laws may require, an insurance plan to include a specific hospital or other facility in their network even at negotiated rates that exceed the OON price cap. Fourth, while OON caps increase the negotiation leverage of insurers, there is no guarantee that those savings will be passed to consumers, especially in markets with dominant insurers. Notwithstanding these potential weaknesses, setting OON price caps may provide a straightforward regulatory intervention that can produce cost savings through altered negotiating dynamics without directly setting in-network price limits. Slowly decreasing the OON cap over time would allow states to monitor the impact of these price caps on quality and access, provide incentives for providers to increase efficiency and reduce costs, and minimize disruptions to the health care delivery system that could result from sudden price reductions (61, 70, 71).

#### Option 3: Affordability standards applied by the state department of insurance

The third “light touch” option is to constrain price *growth* by setting standards for “affordable” premium *increases* approved by the state DOI. We consider affordability standards a light-touch option because they incrementally build on existing authority and can leverage expertise in an existing state agency. Currently, the authority of state DOIs to review insurance premiums falls into two broad categories: states with “prior approval” authority that requires insurers to file plan rates and supporting documentation and get approval from the state DOI before selling plans in the market, and states with “file and use” authority that requires insurers to file proposed rates but allows them to sell health insurance plans without further review (72). The DOI typically ensures that proposed insurance rates are adequate to cover expenses or losses and not unfairly discriminatory, but they typically do not review underlying reasons for proposed rate increases (73). The Affordable Care Act (ACA) requires insurers in the individual and small group markets to *justify* the reasonableness of any increases in premiums above 15% (74), but it does not give regulators the authority to *reject* rate increases that they find to be excessive or unjustified.

A few states go beyond these ACA requirements to authorize the insurance commissioner to reject insurance plans with provider rate increases that exceed a threshold, typically indexed to economic growth (such as the Gross State Product) or a measure of provider input cost increases (like Medicare's hospital Market Basket Index – MBI or professional Medical Expenditure Index - MEI). When using rate review to restrict provider rate increases, state policymakers may set a maximum premium increase deemed “affordable” by regulation and then set explicit limits on annual contracted price updates for providers in those plans. Policymakers should set limits on allowed price increases at the provider (or provider group) level so that providers with more market power cannot demand higher rate increases thereby causing insurers to impose lower rate increases on other providers to comply with an aggregate allowed premium increase. The ability of the state to restrict negotiated price increases can give commercial insurers more negotiating leverage as providers know the maximum insurance rate increases allowed by state law.

##### States' experience with insurance affordability standards

Three states – Rhode Island, Colorado, and Delaware – allow their state insurance commissioner to reject unaffordable premium increases [(75, 76), p. 19]. The affordability standards in Delaware and Colorado are too recent to assess whether they have effectively controlled costs, but Rhode Island has successfully applied affordability standards for more than a decade. Beginning in 2010, the Office of the Health Insurance Commissioner (OHIC) in Rhode Island required all commercial insurers licensed by the state to increase spending on primary care, increase bundled payments and value-based care, and limit individual hospital rate increases to an inflationary benchmark. Specifically, OHIC must approve of any contract if either 1) the average rate increase, including estimated quality incentive payments, is greater than the US Urban Consumer Items Less Food and Energy (CPI-Urban) percentage increase plus one percent, or 2) less than fifty percent of the average rate increase is for expected quality incentive payments (77).

When researchers analyzed spending growth for commercial hospital claims in Rhode Island following the implementation of the affordability standards, they found a reduction in total spending on claims-based hospital care relative to a national control cohort (78). The researchers also concluded that the price growth limits shifted the negotiating dynamics between commercial insurers and hospitals in favor of insurers. However, because the rate update method in Rhode Island examines only percentage increases in hospital rates, it may exacerbate existing inequities in payments because an allowed increase based on current prices allow high-priced hospitals to secure higher absolute dollar increases. Rhode Island recognized this limitation and in 2020, OHIC modified the hospital affordability standards and gave hospitals with below-median prices the opportunity to increase their base rates if they met specific quality requirements. Furthermore, because the price growth limits only applied to hospitals, it may have stimulated physician consolidation (79) and an increase in physician prices in recent years (80). Despite the limitations of this model, the Rhode Island experience shows affordability standards can help moderate the growth in negotiated hospital prices without the need to develop an additional regulatory oversight bureaucracy.

##### Considerations for states implementing affordability standards

State lawmakers may find passing legislation to expand the scope of DOI review of health insurance plans to include an assessment of “affordability” easier than other provider rate regulation models, especially in states with prior approval authority. Furthermore, Rhode Island's affordability standards have successfully kept hospital costs to be some of the lowest in the country (17), and this accomplishment makes the affordability standards model one of the few models with demonstrated success. Additionally, since the affordability standards are applied to the growth in insurance premiums, insurers are required to pass much of the cost savings resulting from lower provider prices to consumers. Finally, this model likely has a lower potential for regulatory capture than the other models because the interests of the DOI and the industry being regulated – the insurers – are aligned. Since the DOI does not directly regulate or regularly interact with representatives of health systems, the risk of regulatory capture of the DOI by providers is lower than for agencies that directly regulate provider prices.

On the other hand, there are limitations to the affordability standards that policymakers should consider when trying to replicate the success in Rhode Island. First, OHIC has the authority to review and approve rates in the individual market, the small-group market, and fully-insured, large-group markets in Rhode Island, but most states do not currently have prior approval authority for large-group plans (72). Importantly, even if lawmakers expand the authority of their DOI to review large group plans, federal ERISA law does not deem self-funded employer plans to be insurance (19). As a result, state affordability standards for health insurance will likely not apply to self-funded employers. Supreme Court decisions in *Rutledge v. Pharmaceutical Care Management Association* (141 S.Ct. 474 (2020)) and *New York State Conference of Blue Cross & Blue Shield Plans v. Travelers Ins*. Co (115 S.Ct. 1671 (1995)) coupled with a history of state hospital rate regulation in the 1980's and 90's (81), suggest that states can likely can pass legislation regulating prices and other cost-control mechanisms as long as they do not regulate a central matter of plan administration (82). Consequently, states with a large portion of residents covered by self-funded plans may be better served by selecting a rate restriction model that applies directly to providers. Nonetheless, states implementing affordability standards may realize some spill-over savings to self-funded products if insurers negotiate rates for both their fully-insured and self-insured products (where they function as third party administrators) at the same time.

Additionally, as mentioned above, policymakers should apply rate increases at the individual insurer-provider contract level and may consider tiered updates where the highest price providers have smaller allowed increases than lower priced providers or a fixed-dollar increase (i.e., not a percentage of previous rates). Policymakers may consider a more complicated formula for rate increases (e.g., allowing a percentage increase for labor costs, but a set dollar increase for fixed costs), but that approach undermines the simplicity of the affordability standard approach. Finally, policymakers should consider applying the affordability standards to all providers (not just hospitals) to minimize the incentives for physician consolidation or the ability of providers to increase prices for services outside the regulation caps.

### Step 4: Evaluate more robust payment models that require significant administrative oversight

Some states may find that the light-touch models are insufficient to constrain price increases or to narrow provider price disparities. Other states may decide that they have the legislative will or regulatory capacity to skip over the lower intensity models and move to rate restriction options that require more significant administrative oversight.

#### Hospital global budgets

Hospital global budgets are a prospectively determined cap on annual revenues where the total budget is set in advance. When using this option, regulators assess regulatory compliance at the aggregate budget level, obviating the need to develop a highly complex system of regulated prices for individual services. Hence, the administrative oversight needed to oversee a global budget is less intensive than traditional rate-setting systems (83). Global budgets are most easily implemented on an all-payer basis, and policymakers typically limit annual growth in the budget to a benchmark, like the gross state product or Medicare's MBI. Budgets can be fixed or semi-variable, where a hospitals receives a base amount for fixed costs and additional payments based on variable costs for provided services. The semi-variable global budget allows the budget to adjust to changes in patient demographics in the hospital's service area or a change in the number of patients served. States can include supplemental rewards and penalties for quality measures like readmission rates, rates of hospital-acquired infections, patient satisfaction scores, and emergency room wait times (84). Both the fixed and semi-variable budget options counteract the incentive to increase care under FFS payment systems, but the semi-variable approach reduces the tendency to inappropriately stint on care, such as increasing queues for care, cutting out less profitable service lines, or rerouting hospital ambulatory care services to unregulated providers (83). Traditionally, hospital global budgets are based on each hospital's historical revenues generated in the most recent year, which can ease the transition to this new payment model.

Global budgets can appeal to hospitals, particularly those in rural areas with shrinking patient volumes, because global budgets guarantee either an annual fixed revenue stream or, in the case of more flexible budgets, sufficient revenue to cover a hospital's fixed costs should volumes decline. Any savings generated by a hospital under this model can be reinvested in care coordination initiatives and activities to help improve the overall health of the populations served by a global budget hospital (85). Hospital global budgets also appeal to payers because regulatory limits on both annual hospital per capita expenditures and expenditure growth can dramatically improve the affordability of hospital care.

##### The experience of states with global budgets

All-payer hospital global budgets (requiring a waiver from Medicare/Medicaid payment rules) have been implemented both on a regional and state-wide level, with varying levels of flexibility. In the 1980's, two separate quasi-public oversight entities implemented global hospital budgets for nine urban hospitals in Rochester, New York, and eight rural hospitals in the Finger Lakes region. These systems placed limits on annual hospital revenue but allowed for changes in annual revenue based on changes in each facility's variable costs. These regional models were less administratively complex than direct rate-setting systems because they were “formula-driven” and applied regulatory constraint at the aggregate hospital budget level rather than regulating the price of each individual hospital service (86). These models demonstrated cost containment success and improved financial performance of hospitals until they were ended in 1987, when the hospitals determined that they could receive more lucrative payment levels under the newly implemented Medicare inpatient prospective payment system (IPPS) system (86, 87).

At a state-wide level, Maryland implemented fixed hospital global budgets for 10 of the state's rural hospitals beginning in 2009 and extended this approach to the remaining 37 acute care hospitals in 2014. Between 2014 and 2018, Maryland's model reduced hospital spending for Medicare, reduced total expenditures for Medicare, reduced admissions for Medicare and commercial payers, and reduced emergency department visits for Medicaid and commercial payers (84). More recently, Pennsylvania obtained a waiver from CMS in 2017 to adopt a global budget payment model for critical access and other acute care hospitals in rural areas, but the model has experienced operational delays and faced implementation challenges due to the COVID-19 pandemic. As a result, it remains unclear if Pennsylvania's model is a viable, long-term solution to support rural hospitals (88).

##### Considerations for states implementing global budgets

To avoid cost-shifting between payers or hospitals, hospital global budgets work best when all payers (both public and private) and all or most hospitals in a state or region of the state to participate on a mandatory basis (83, 89). If some payers, especially large payers, do not participate, there could be windfalls or shortfalls to participating payers as volumes fluctuate. Furthermore, all-payer models apply uniform financial incentives and can be constructed to reduce the possibility of cost-shifting from one payer to another. As a result, states should obtain a waiver from CMS to ensure both Medicare and Medicaid participation in a global budget payment model. The current gulf between commercial rates and Medicare rates means that states looking to implement a global budget are unlikely to obtain a waiver to equalize Medicare, Medicaid, and commercial payment levels like the one approved for Maryland in 1977 (90). However, Pennsylvania's more recent waiver allowed the state to include Medicare and Medicaid in their rural global budget model and control the rates of growth of payment levels from status quo levels (88). Other states could reasonably expect to obtain a similar waiver from CMS if the rate model could ensure some future savings to the federal government, the approach adopted in Pennsylvania and Vermont (which received a waiver for its all-payer ACO model).

To help align incentives between physicians and hospitals under global budgets, states may choose to include payments to hospital-employed physicians and hospital-owned physician practices. States may also consider implementing supplemental pay-for-performance incentive programs for global budget hospitals to help maintain or improve quality of care. Finally, under fixed global budgets, hospitals will have incentives to shift care to other global-budget hospitals or providers not covered by the global budget. Flexible global budgets can reduce the incentives to shift care to other provider and an additional adjustment mechanism can be applied to either fixed or flexible global budgets to further address this issue. However, such an additional adjustment can add to the complexity of this model.

Global budgets may be an attractive option for states looking to move beyond the low-intensity regulatory models as they have demonstrated cost containment success and improved financial performance of hospitals, previously in New York and currently in Maryland. While a regulatory agency is necessary to oversee global budgets, the level of oversight can be much lower than for an agency that applies price caps on all services or regulates rate updates. The focus on the aggregate budget makes the regulatory process simpler and various adjustments (such as the use of aggregate stop-loss provisions) can ensure the financial stability of any hospital, including critical access hospitals and other rural facilities. A state could choose to phase implementation by starting with rural hospitals and including more facilities over time as it gains experience setting and updating global budgets. Starting with rural facilities, where patients are primarily treated at one hospital, can also facilitate the development of a more regional approach to cost containment through the inclusion of hospital employed and other services (such as post-acute, home health and long-term care) in the global budget process and align incentives of most area providers in controlling costs (89). Hospital global budget models can also allow a state to support all hospitals during periods of untoward circumstances such as was the case in Maryland during the COVID-19 pandemic (91).

#### Capping all service prices (both in- and out-of-network) and regulating annual price updates

An alternative to establishing hospital global budgets is for a state agency to directly set prices for all healthcare services. In this section, we discuss three related ways that a state might implement such direct price regulation: (1) price setting, where a state agency establishes a specific price for all healthcare services, (2) capping prices, where a state agency places a ceiling on prices and allows negotiation below that cap, and (3) capping price *increases*, where a state agency limits how much provider prices can increase over a defined period. If the price increases are graduated or tiered such that highest priced providers are allowed smaller price increases, capping price increases can cause convergence of prices over time and eventually result in a system that mirrors price caps. If a state establishes prices or price caps, policymakers will need the regulatory authority to update those levels year over year. As a result, these options all require establishing a state agency with the authority to set and update payment rates and require compliance with the regulated prices/price caps.

Many economists have projected significant savings to the U.S. healthcare system if there was a nationwide cap on prices between 100 and 300% of Medicare FFS rates (92–94). While some rate setting proposals have suggested setting prices or price caps at a multiple of Medicare rates (83, 95), several prominent health economists proposed setting caps at the very top of the price distribution based on local market levels, i.e., five times the 20^th^ percentile of prices at the local market level (48). Their proposal also includes tiered caps on price growth to reduce the price variation by provider and gradually converging prices over time. While the proposal by Chernew et al., was intended to minimize market disruption and administrative complexity by setting relatively high caps an allowing the market “room to work” under those caps, setting caps on all hospital prices along with limits on each provider's allowed price updates will likely require extensive regulatory authority and infrastructure. Thus, we consider this model a more complex and intensive regulatory approach.

##### The experience of states with caps on all prices and regulated rate updates

During the 1960's and early 1970's, as many as 30 states implemented state-based programs to either review or regulate hospital rates and budgets (81). Of these, seven states adopted mandatory pricing models in which state law required the participation and compliance of all hospitals. These mandatory state rate-setting systems demonstrated some success in controlling hospital prices and per capita expenditures (96–98). States looking to set price caps in the commercial market may look to West Virginia's rate regulation model, which ran from 1990 to 2016 (99). Under this system, the West Virginia Health Care Authority set both an upper rate limit and a rate floor for all hospital service prices each year. Hospitals and insurers were then free to negotiate annual prices within these pre-established and regulated pricing corridors. The methodology also applied tiered rate updates to each hospital's upper rate limit based on the relative priciness of each hospital (i.e., higher priced hospitals received lower annual updates). The upper rate limit protected insurers from the ability of dominant hospitals to demand very high commercial prices, whereas the floor was intended to protect hospitals from dominant insurers that are able to use their market power to push rates below average cost (96). This corridor approach should allow market forces to control costs within government applied guardrails, but the West Virginia rate-setting system had only modest reductions in costs (96, 98). Because the system failed to adequately constrain both volumes and prices, per capita hospital spending in West Virginia was quite high, and the legislature terminated the hospital rate setting system in 2016 (96).

Rather than setting rates, Massachusetts proposed regulating price increases for hospitals. The state considered establishing three tiers of allowed price increases where hospitals were grouped based on each facility's blended inpatient and outpatient relative price levels (100). In addition to gradually compressing hospital price levels over time, this bill would have limited overall rate updates such that premium increases were consistent with the state's CGB. While this bill failed to pass, it demonstrates that policymakers are considering ways to establish allowed price increases as one mechanism to control costs.

##### Considerations for states implementing caps on all prices and regulated rate updates

States considering establishing prices or price caps with regulated price updates should not underestimate the difficulty in developing the elaborate data, administrative, analytic, and regulatory oversight infrastructures to establish, adjust, monitor, and regulate prices over time for all services, while accounting for regional differences and variations in costs. Such a model will require extensive and detailed data collection on a monthly or quarterly basis to monitor and enforce hospital compliance with established caps and price updates. These significant regulatory requirements and detailed and complicated cap setting/cap update methodology may make this model susceptible to regulatory capture and failure, so policymakers should consider ways in which these vulnerabilities can be minimized (52).

In addition to the regulatory burden, policymakers should recognize that these rate-setting models give providers a financial incentive to increase the amount of care delivered and that it is difficult incorporate APMs, like capitated or risk-based contracts, into these models. Chernew et al. acknowledge that APMs “can serve as a vehicle to circumvent regulation of fee-for-service prices” and suggest that a state agency be tasked with authority to address potential circumvention, adding to the complexity of the rate-setting model (48). Accordingly, states may wish to experiment with the lighter touch options and turn to this highly regulatory system if those other methods prove ineffective.

#### Population-based payment model

States looking to address the inherent limitations of rate setting agencies may consider a Population-Based Payment (PBP) model. PBPs aim to create a highly integrated finance and delivery system to meet population-level cost and quality targets. These systems give health care providers a spending target for the care of a defined patient population and are structured to provide financial incentives to encourage providers to deliver well-coordinated, high-quality, and person-centered care. Three key features characterize PBP models: (1) they are prospective, with payments to all providers constrained by an overall budget and require providers to take on risk for costs of care that exceed the budgeted amount; (2) they require patient attribution, a provider organization is accountable for the cost and quality of care delivered to a specific population; and (3) they allow provider organizations to proactively manage care and costs for the covered population.

These systems usually go beyond establishing global budgets covering hospital and hospital-based physicians to extend budget-based payment incentives to all physician care and cover a wide range of preventive health, care coordination, and wellness services. The model replaces the volume-based incentives of FFS payment models with payment that incentivizes providers to deliver necessary services that help improve care quality and the population's health status covered by the model (101).

##### State experience with PBP models

Vermont tried to establish a PBP system through a state-wide, all-payer ACO called OneCare that created overall budgets for hospital and non-hospital health expenditures on a regional basis. OneCare contracts with Medicare, Medicaid, and Blue Cross Blue Shield of Vermont, the largest commercial insurer in the state and relies on voluntary participation by providers and commercial payers. Under the waiver granted by CMS, Vermont committed to limiting all-payer Total Cost of Care (TCOC) annual growth to 3.5% and Medicare TCOC growth to 0.2% below the national Medicare growth rate (102). During the model's first 3 years of operation, it decreased hospital-based utilization and expenditures for Medicare, but the model failed to meet participation targets for Medicare beneficiaries and commercially insured patients, especially patients with coverage through self-funded employers (103). Smaller rural hospitals have also been reluctant to participate in the model due to the financial risk involved (103).

Vermont is a small, relatively rural state, so it is well-suited to develop a PBP model. Specifically, the state's 14 distinct geographic regions and populations are each served by one hospital and other providers, many of which have ties to the regional hospital, so attributing TCOC payments for a region to a hospital is relatively straightforward. However, the Vermont experience demonstrates the various challenges associated with developing a PBP, including: (1) developing the required level of clinical and financial (through risk sharing) integration necessary to be successful through the use of an ACO structure; (2) ensuring the participation of all hospitals, physicians, other providers (such as Federally Qualified Health Centers) and payers, particularly ERISA plans, which may be exempt from any state mandate to participate in the model; and (3) the difficulties of a voluntary model in which core participants may exit if they determine the model does not serve their strategic and financial interests (103).

##### Considerations for states implementing PBPs

Theoretically, a PBP could provide a state with the most effective model for overall cost control, covering a majority of services and provider types, along with opportunities to include incentives for providers to improve overall care quality and population health, but it remains to be seen whether individual states can successfully implement such a comprehensive and highly integrated care management and cost containment approach. Future iterations may incorporate elements of other PBP-like models such as Alain Enthoven's Managed Competition model which was extensively discussed at a national level in the 1990's (104) or the possible expansion of MA to include commercially insured members (105). Such an approach might create a chassis for states to use to develop a PBP-based model for a broad proportion of their populations. Yet, in the absence of these developments at the federal level, states will likely encounter difficulties in developing PBP models outside of payer-specific PBP models, such as the Massachusetts BlueCross BlueShield Alternative Quality Contract (106).

## Design considerations

When assessing the spectrum of options, state policymakers likely want to consider characteristics of the state including the rural and urban make-up of the state (e.g., does one hospital provide most of the care for most residents in some areas), the administrative resources available to the state, and the political environment. After selecting one or more models to pursue, policymakers should ask a few guiding questions to help design their chosen model in a way that maximizes its effectiveness.

**What methodology should be used to set prices?** When implementing any of these models, policymakers may choose to benchmark commercial rates to those set by public payers (i.e., a percentage of Medicare rates) or to some measure of commercial rates (i.e., the median in-network rate). Medicare prices are adjusted for legitimate cost differences across providers and give providers a transparent benchmark and actionable information when trying to reduce costs. A few states, including Montana and Oregon, successfully base the rates their SEHBPs pay hospitals for both in-and OON coverage to a multiple of Medicare prices, saving both states millions of dollars (107). On the other hand, some Medicare prices are distorted by political forces resulting in overpayment for some specialties and underpayment for others, including primary care (48, 108, 109). For example, many experts think the Relative Value Update Committee (RUC), which surveys physicians to set relative payment rates for physician services, has been excessively influenced by specialists which dominate the decisions of the RUC (108, 110). Furthermore, using Medicare rates to benchmark commercial payment rates may increase pressure on Congress to raise Medicare prices (48). Economists studying the passage of the 2003 Medicare Modernization (MMA) found that lobbying by the healthcare industry likely led to higher Medicare payments as politicians used Medicare payment increases as a tool to win votes from members of Congress and members of Congress representing hospitals that got a payment increase received large increases in campaign contributions before and after the program was extended (111). Such political pressure would likely increase if Medicare rates were used as a benchmark to set provider rates. In addition, tying state payment rates to Medicare rates could complicate Medicare payment polices (e.g., the Medicare Sequester, productivity adjustments, MACRA physician fee updates) (48).

Alternatively, some experts proposed setting price caps relative to median commercial prices, arguing that indexing commercial rates to Medicare rates adopts all of the known distortions and political forces that complicate Medicare rate setting (48). But distortions, market power, and political forces exist in commercial prices as well. Economic studies demonstrate very high prices for commercial insurance relative to Medicare and substantial heterogeneity in prices across regions, across hospitals within a region, and even within a hospital for different payers (17, 112). Consequently, using existing commercial prices as a benchmark perpetuates status quo prices largely driven by provider negotiating leverage and not relative cost or quality differences (4). Additionally, calculating benchmarks based on in-network commercial rates in a geographic area requires extensive data collection and raises questions about how this standard is calculated (i.e., is a true median or a weighted average, etc.). While Chernew et al., propose setting rates at five times the 20^th^ percentile price to try to minimize problems with outlier prices or data noise (48), the more fundamental problem of obtaining reliable data for these calculations may require federal action as states are unable to compel self-funded plans to report claims data to APCDs.

**How can prices be adjusted over time?** When using either Medicare or existing commercial rates as a benchmark, policymakers likely want to set initial prices near current rates and constrain future growth and/or ratchet them down gradually over time to minimize significant market impacts when the pricing model is adopted (89, 113). This option gives policymakers the ability to phase in regulatory constraints and monitor impacts on quality, provider financial condition, stability of the delivery system, etc. Some experts have argued that imposing a relatively high price cap poses a smaller risk of adverse effects on access and quality and gives market participants “room to work,” which may be particularly valuable if other procompetitive reforms are implemented (48). Accordingly, policymakers may want to start with price limitations that affect the highest priced providers and authorize further price restrictions if savings targets are not met.

Additionally, all of these models have the potential to drive prices below costs, leading to unsustainable financial losses (as occurred in the New York State hospital rate setting system in the 1970s and 1980s, leading to significant operating losses for most hospitals in the state). Models that reduce prices over time, allowing providers to adjust to those constraints and tiering price growth, such that the providers with the lowest prices are allowed larger rate increases than the highest-priced providers, will improve payment equity and reduce the likelihood that rural or other safety-net providers will be adversely affected (48). Nonetheless, policymakers concerned about access in rural or low-income areas may also want to consider price floors to protect their solvency and alleviate quality-reduction concerns.

**How can policymakers minimize the likelihood of regulatory capture or failure?** Policymakers will likely want to minimize government interference with the essential activities and decision-making of providers, payers, and patients because complex rate-setting systems with broad regulatory authority and extensive regulatory infrastructures can be prone to regulatory failure or capture by the industry they are intended to regulate (52, 81). Future regulatory pricing systems should be as predictable and manageable as possible, require minimal governmental infrastructure to oversee the system, and ensure compliance with the established price and revenue constraints. Further, states can impose regulatory structures (e.g., establishing a politically independent agency, using leadership with no affiliation to the regulated industry, strict use of a state's Administrative Procedures Act, etc.) that can help minimize the potential for both capture and failure (52).

**How do provider price restrictions affect competition in the insurance market?** These models give policymakers a way to restrict prices for healthcare services. Restricting prices directly allows self-funded employer-based plans to realize any savings directly, but individuals and employers purchasing fully-funded plans rely on competition in the insurance market to compel insurers to pass on the savings through lower premiums and cost-sharing reductions (8, 9).

Perhaps surprisingly, limited evidence suggests that rate regulation models can stimulate market entry of new insurers. The need to immediately construct a robust provider network creates a significant barrier to entry for new insurers, but if a state has pricing restrictions, the financial exposure of an insurer is limited if enrollees obtain care from out-of-network providers. Thus, OON caps or other price regulation may reduce the financial consequences of a limited network and make it easier for smaller managed care plans to develop viable networks at reasonable prices. Limited evidence suggests that price caps have had this effect: two insurers entered the individual market in Washington state following price caps in the public option (49, 114) and Massachusetts, Maryland, and New York experienced above-average entry of insurers during the years they had rate setting systems in place (81).

Nonetheless, it remains unknown if rate regulatory models will induce sufficient competition in the insurance market to allow consumers to realize savings from reduced provider prices. The ACA places restrictions on profits that insurers can generate by requiring plans to spend a minimum percentage of premiums on medical care. Individual and small group insurance plans must have an annual minimum medical loss ratio (MLR) of 80% and large group plans must have a minimum MLR of 85% or the insurer must give rebates to policyholders (115). The MLR requirements provide insurers an incentive to increase payments as the profit and administrative fees the insurer collects are based on the total paid on claims. The MLR requirement can be a double-edged sword as insurers have a smaller incentive to reduce prices as they can generate larger profits by paying more in claims (116). On the other hand, if provider prices are restricted by state policies, the MLR requirement on insurers can help ensure that those savings are passed on to consumers.

### Summary of considerations for states choosing potential options

When considering rate regulation models, all states should then begin with an assessment of healthcare markets in the state and consider factors related to health care costs including: (1) the level of overall commercial prices as a proportion of comparable Medicare prices, (2) recent rates of price and expenditure growth by provider sector; (3) recent price growth and per capita expenditure growth trends by provider sector; (4) the level of existing pricing disparities among providers serving different patient populations or serving different regions;(5) levels and rates of provider consolidation by provider sector; and (6) the level of commercial insurer consolidation. As part of this process, policymakers may establish a CGB to give policymakers a better understanding of the underlying dynamics of state healthcare markets. Then policymakers likely want to assess the potential impact of a light-touch, low-regulatory intervention model. Starting with a public option, OON caps, or affordability standards allows the state to gain experience evaluating appropriate prices and variances in prices across the state and among different providers. States seeking more robust provider rate restrictions likely want to consider a flexible, hospital global budget model. Policymakers may choose to start implementation on a smaller basis (i.e., with rural hospitals or only hospital-based care) and expand. Policymakers may also consider capping prices for all health care services and establishing rate updates, and policymakers seeking the highest level of control over health care expenditures may want to explore the development of a PBP model. With any of the models, policymakers should consider how to design the policy to control costs in addition to prices and how to establish rates in a manner that minimizes the potential for regulatory capture and ensures that consumers benefit from lower prices.

## Conclusion

Spending on health care crowds out spending on other priorities (48, 117) and suppresses wage growth for American workers (118). With Congressional action highly unlikely, the biggest danger faced by state policymakers is inaction. State lawmakers have a spectrum of options that can be adopted and tailored to key policy goals, specific market conditions, and the political environment of the state. In this report we describe the experiences of forerunner states and offer considerations for other states seeking to learn from them. It remains unknown whether the relatively incremental interventions–the public option, OON caps, and affordability standards–can adequately address specific market inefficiencies or market failures to control prices or whether the more administratively complex models–global budgets, caps on prices and price updates, or PBPs–can promote or redirect competition to dimensions other than costs, like quality and patient experience (83). Nonetheless, there is a pressing need for lawmakers to act. Rather than passively considering options, policymakers should choose the rate regulation models that best fit their goals and political climate and adapt them as market conditions change.

## Author contributions

KG and RM conceived of the work, collected the research, drafted the manuscript, and revised the manuscript for important intellectual content. Both authors have read and approved the final manuscript.

## Funding

This study was funded by the Laura and John Arnold Foundation.

## Conflict of interest

Authors KG and RM are employed by the Source on Healthcare Price and Competition at the University of California, Hastings College of the Law. Author RM is also employed by Global Health Payments LLC.

## Publisher's note

All claims expressed in this article are solely those of the authors and do not necessarily represent those of their affiliated organizations, or those of the publisher, the editors and the reviewers. Any product that may be evaluated in this article, or claim that may be made by its manufacturer, is not guaranteed or endorsed by the publisher.

## Author disclaimer

The opinions, results, views, and conclusions reported in this article were those of the authors and do not necessarily reflect those of our funders.
